# VHL-deficient renal cancer cells gain resistance to mitochondria-activating apoptosis inducers by activating AKT through the IGF1R-PI3K pathway

**DOI:** 10.1007/s13277-016-5260-2

**Published:** 2016-07-26

**Authors:** Ryuji Yamaguchi, Hiroshi Harada, Kiichi Hirota

**Affiliations:** 1 Department of Anesthesiology, Kansai Medical University, 2-5-1 Shinmachi, Hirakata, Osaka, 573-1010 Japan; 2 Department of Radiation Oncology and Image-applied Therapy, Kyoto University Graduate School of Medicine, Kyoto, 606-8507 Japan; 3 Precursory Research for Embryonic Science and Technology (PREST), Japan Science and Technology Agency (JST), Saitama, 332-0012 Japan

**Keywords:** 2-deoxyglucose, ABT-263, Apoptosis, PI3K, Ras, ERK1/2, AKT

## Abstract

We previously developed (2-deoxyglucose)-(ABT-263) combination therapy (2DG-ABT), which induces apoptosis by activating Bak in the mitochondria of highly glycolytic cells with varied genetic backgrounds. However, the rates of apoptosis induced by 2DG-ABT were lower in von Hippel-Lindau (VHL)-deficient cancer cells. The re-expression of VHL protein in these cells lowered IGF1R expression in a manner independent of oxygen concentration. Lowering IGF1R expression via small interfering RNA (siRNA) sensitized the cells to 2DG-ABT, suggesting that IGF1R interfered with the activation of apoptosis by the mitochondria. To determine which of the two pathways activated by IGF1R, the Ras-ERK pathway or the PI3K-AKT pathway, was involved in the impairment of mitochondria activation, the cells were treated with a specific inhibitor of either PI3K or ERK, and 2DG-ABT was added to activate the mitochondria. The apoptotic rates resulting from 2DG-ABT treatment were higher in the cells treated with the PI3K inhibitor, while the rates remained approximately the same in the cells treated with the ERK inhibitor. In 2DG-ABT-sensitive cells, a 4-h 2DG treatment caused the dissociation of Mcl-1 from Bak, while ABT treatment alone caused the dissociation of Bcl-xL from Bak without substantially reducing Mcl-1 levels. In 2DG-ABT-resistant cells, Mcl-1 dissociated from Bak only when AKT activity was inhibited during the 4-h 2DG treatment. Thus, in VHL-deficient cells, IGF1R activated AKT and stabilized the Bak-Mcl-1 complex, thereby conferring cell resistance to apoptosis.

**Electronic supplementary material:**

The online version of this article (doi:10.1007/s13277-016-5260-2) contains supplementary material, which is available to authorized users.

## Introduction

A single cancer cell that remains after surgery and/or chemotherapy can cause cancer recurrence [1]. Therefore, cancer chemotherapy aims to eliminate all cancer cells. Given the heterogeneity of cancer cells in a tumor, it is difficult to eliminate all cancer cells using a single agent that targets a particular gene product [2]. To develop a therapy capable of specifically inducing apoptosis in cancer cells across varied genetic backgrounds, we targeted cells with elevated glucose metabolism. In the body, such cells are present in inflamed tissues, muscle cells under heavy exertion, cancer cells, and brain cells and are precisely the cells identified by FDG-PET scans. To target these cells, we previously developed the combination therapy 2-deoxyglucose-ABT-263 (2DG-ABT) [3]. When 2DG is transported across the plasma membrane through the glucose transporter, it is converted into 2DG-6-P by hexokinase in the cytosol and becomes trapped in the cell. Because 2DG-6-P is a poor substrate for glucose-6-phosphatase, it accumulates in the cytosol, competitively inhibiting hexokinase activity. As early as in 1954, 2DG was tested in mouse models of cancer [4]. However, at the pharmacological dose of 40 mg/kg, 2DG only delays tumor growth. Thus, more recently, 2DG has been evaluated in combination with other chemotherapeutics [2]. When 2DG-6-P accumulates in the cell, it causes the dissociation of the anti-apoptotic protein Mcl-1 from the mitochondria, usually within 2–4 h, thus sensitizing cells to mitochondria-dependent apoptosis. The precise molecular mechanism of how 2DG induces the dissociation of Mcl-1 from Bak is not known [2].

ABT-263 (ABT) is a potent, orally bioavailable Bcl-2-family inhibitor [5]. ABT binds specifically and with high affinity to the Bcl-2 family of proteins, including Bcl-2, Bcl-xL, and Bcl-w, but not Mcl-1, and releases these proteins from the mitochondria [6]. ABT has limited use when used as a single agent in cancer therapy; however, when used with other chemotherapeutics, it can enhance their efficacies against hematologic tumors [7]. Recent evidence has suggested that unlike 2DG, ABT can target quiescent cells. Additionally, ABT has been observed to target and clear senescent hematopoietic cells in progeria mouse models, rejuvenating their aged hematopoietic system [8].

When cells are exposed to both 2DG and ABT, the pro-apoptotic Bak and Bax proteins are freed from all their inhibitory associations with Mcl-1 and the rest of the Bcl-2 family of proteins and become activated, releasing cytochrome c from the mitochondria. The release of cytochrome c triggers the formation of the apoptosome in the cytosol. The formation of the apoptosome, often called the death executioner, and the release of cytochrome c are often called the points of no return. Thus, unlike other initiators of apoptosis, 2DG-ABT induces apoptosis very quickly, circumventing the many steps that are usually required and leading to the point of no return within a few hours. As a single agent, neither 2DG nor ABT is very effective [2]. However, when cells take up both agents, the two agents synergize and induce apoptosis. Because ABT cannot cross the blood-brain barrier, it accumulates in tissues outside the brain. Thus, the 2DG-ABT combination therapy induces apoptosis in cancer cells outside the brain [2]. The 2DG-ABT combination has been shown to efficiently induce apoptosis in p53-compromised, PTEN-deleted, highly chemo-resistant human prostate cancer cells xenografted into mice [3].

When 2DG is injected into the body, it accumulates in the bladder within 4 h, and less than 50 % of the drug is found in circulation. To mimic the environment of cancer cells in the body, we used the following protocol for in vitro experiments: 10 mM 2DG was added to culture medium containing 25 mM glucose, and ABT-263 was added 2 h later; the cells were washed 4 h after 2DG addition, after which, they were incubated in fresh medium. We previously showed that when 2 mM 2DG is present in medium containing 5 mM glucose, it synergizes with ABT [3]. However, because our cells grew substantially faster in high-glucose medium, we used a 10 mM 2DG/25 mM glucose combination. Using this protocol, when 2DG-ABT was tested on various cancer cells in vitro, it induced apoptosis in all types of cancer cells across varied genetic backgrounds. However, its efficiency varied among cell lines. For example, as a single agent, 2DG-ABT induced apoptosis in over 94 % of MCF-7 breast cancer cells and PPC-1 prostate cancer cells [3]. However, using an identical protocol, the apoptotic rates were well below 50 % in many renal cancer cell lines. Accordingly, we investigated the reason underlying resistance in renal cancer cell lines.

## Results

### The rates of apoptosis induced by 2DG-ABT were lower in VHL-deficient cancer cells under both normoxic and hypoxic conditions

**Fig. 1:**
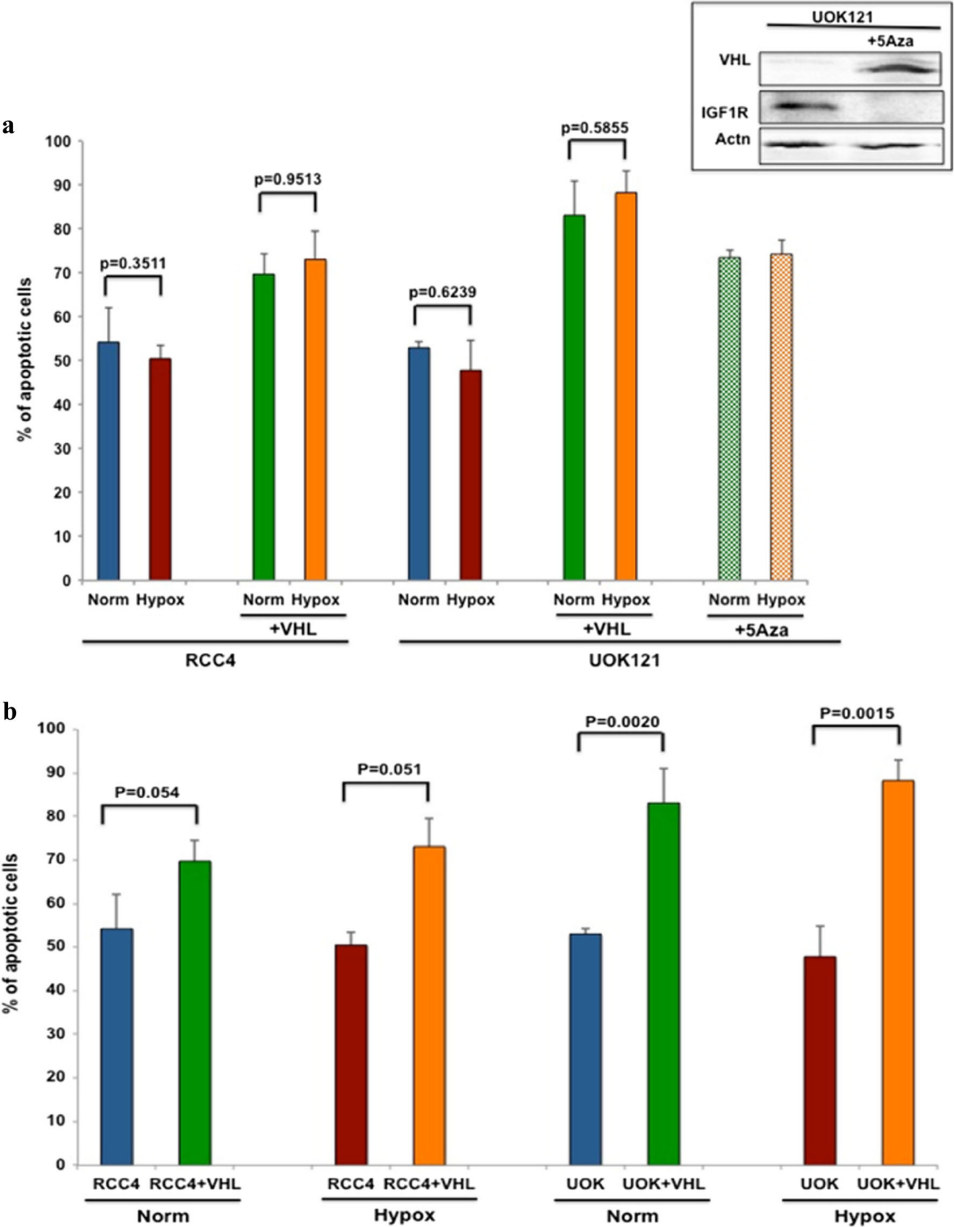
The rates of apoptosis induced by 2DG-ABT were lower in VHL-deficient cancer cells under both normoxic and hypoxic conditions. **a** The renal cancer cell lines, RCC4, RCC4 + VHL, UOK121, UOL121 + VHL, and UOK121 were treated with 5-Aza-dC (UOK121 + 5-Aza-dC) to restore VHL expression. Subsequently, the cells were tested for sensitivity to 2DG-ABT combination therapy. First, the cells were treated with 10 mM 2DG and, 2 h later, with 10 mM ABT. At 4 h from the start of the combination treatment, the cells were washed and re-incubated in fresh media. Cells were assayed under either normoxic (21 % oxygen) or hypoxic (1 % oxygen) conditions and analyzed for propidium iodide incorporation by FACS. Graphs of the FACS data are shown. Inserted Panel. Western blots of UOK121 cells treated with or without 5-Aza-dCyd. **b** For ease of comparison, the rates of apoptosis of cells with or without VHL were placed next to each other in this panel

To improve the efficacy of 2DG-ABT combination therapy, we investigated the cause of cancer cell resistance to 2DG-ABT treatment. We found that the renal carcinoma cell line RCC4 was moderately resistant to 2DG-ABT. In vitro, ABT induced apoptosis in less than 20 % of these cells, even at a 10 μM concentration. However, when ABT was combined with 2DG, the combination induced apoptosis in 25 % of RCC4 cells at 1 μM ABT and in 55 % of cells at 10 μM ABT (Sup Fig. 1). Given that 2DG combined with 1 μM ABT has been shown to induce apoptosis in over 94 % of PPC-1 prostate and MCF7 breast cancer cells [3], there must be a resistance mechanism present in RCC4 cells. Because RCC4 is deficient in the von Hippel-Lindau (VHL) tumor suppressor gene, which is part of the oxygen sensory/response pathway, hypoxia-inducible factor 1 (HIF1a) is constitutively activated in RCC4. Thus, we tested whether the HIF1a activation would cause RCC4 cell resistance to 2DG-ABT. To test this hypothesis, in addition to the renal cell carcinoma cell line RCC4, we used another renal cancer cell line, UOK121, in which VHL is epigenetically silenced, along with the RCC4 + VHL and UOK121 + VHL cell lines, which were stably transfected with functional VHL. We also treated UOK121 with 5-aza-2′-deoxycytidine (5-Aza-dCyd) for 10 days to demethylate its genomic DNA and restore VHL expression [9]. All cells were assayed under normoxic (21 % oxygen) and hypoxic (1 % oxygen) conditions, and the results are shown in Fig. 1. The presence of VHL in RCC4 and UOK121 cells restored sensitivity to 2DG-ABT induced apoptosis (Fig. 1b: unpaired *t* tests; *p* = 0.054 for normoxic RCC4 cells, *p* = 0.051 for hypoxic RCC4 cells, *p* = 0.0020 for normoxic UOK121 cells, and *p* = 0.0015 for hypoxic UOK121 cells). Furthermore, UOK121 cells that were treated with 5-Aza-dCyd to induce VHL expression were also sensitized to 2DG-ABT (compare the bars representing UOK121 with the bars representing UOK121 + 5Aza in Fig. 1a).

**Fig. 2:**
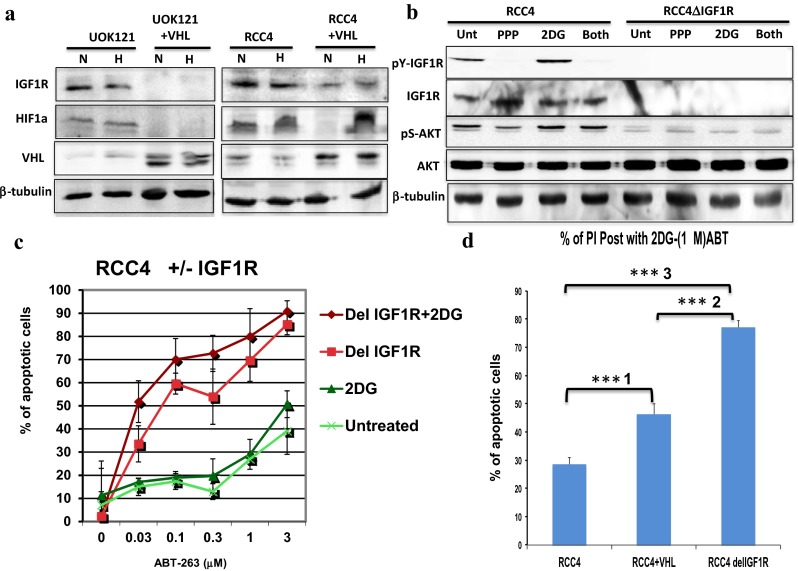
The absence of VHL stabilized IGF1R expression independent of oxygen concentrations and interfered with mitochondria-dependent apoptosis. **a** Western blots of RCC4, RCC4 + VHL, UOK121, and UOK121 + VHL cells cultured under normoxic (N) or hypoxic (H) conditions and probed with antibodies against IGF1R, Hif1a, VHL, and beta-tubulin. **b** Western blots of either RCC4- or IGF1R-depleted RCC4 (RCC4ΔIGF1R) cells treated with 2DG, the IGF1R specific inhibitor, picropodophyllin (PPP), both, or left untreated. The blots were probed with antibodies against phosphor-tyrosine-IGF1R, phosphor-serine473-AKT, pan-IGF1R, pan-AKT ,and β-tubulin. **c** FACS analysis of RCC4- and IGF1R-depleted RCC4 cells treated with 0–3 mM ABT, with or without 2DG. **d** Comparison of apoptotic rates using 10 mM 2DG and 1 mM ABT-263 in RCC4 (28+/−3 %), RCC4-VHL (46.206 %+/−3.91) and RCC4 cells in which IGF1R had been depleted, designated RCC4 delIGF1R (77+/−2). The unpaired *t* test results were *1*p* = 0.0052, *2*p* = 0.0024, and *3*p* = 0.0003. We noted that RCC4 delIGF1R was even more sensitive than RCC4 + VHL, in which there is some IGF1R expression

Interestingly, whether the experiment was performed under normoxic or hypoxic conditions had little effect on the outcome (Fig. 1a: *p* values for unpaired *t* test varied from 0.3511 to 0.9513). Furthermore, HIF1a expression did not influence the sensitivity of the cells to apoptosis. For example, the sensitivities of RCC4 + VHL cells under hypoxia, and thus expressing HIF1a, and RCC4 + VHL cells under normoxia, and thus not expressing HIF1a, to 2DG-ABT at 10 μM ABT-263 were approximately the same (unpaired *t* test *p* = 0.9513 and HIF1a blots are in Fig. 2a). Thus, the presence of VHL influences cellular sensitivity to apoptosis, but not necessarily by activating the HIF1a transcription factor. We also noted that in VHL-expressing UOK121 cells, there was no HIF1a expression, even under hypoxic conditions (Fig. 2a), most likely because the over-expressed VHL degrades HIF1a, even under hypoxic conditions [9]. The results of more extensive testing of the 2DG-ABT combination using varying concentrations of ABT from 0 to 10 μM are reported in Sup Fig. 1, and the statistical analysis is shown in Sup Table 1. The results showed that the addition of 2DG synergized with ABT, thereby increasing the apoptotic rates in RCC4 cells with or without VHL expression and in UOK121 cells with VHL expression, as expected from previous studies [3, 10].

### The absence of VHL stabilized IGF1R expression independently of oxygen concentration and interfered with mitochondria-dependent apoptosis

We searched the literature and databases for genes regulated by VHL independent of oxygen concentration and found that IGF1R is up-regulated in the absence of VHL, regardless of the oxygen concentration. Yuen and colleagues found that IGF1R protein levels are unaffected by hypoxia in clear cell renal carcinoma with or without VHL, but exogenously introduced VHL protein reduces both the promoter activity of IGF1R and the stability of IGF1R mRNA independent of oxygen concentration [11]. We independently verified that IGF1R protein levels decreased when the VHL protein was introduced into UOK121 and RCC4 cells (Fig. 2a). When we depleted IGF1R from RCC4 using siRNA, we observed an increased sensitivity of the cells to 2DG-ABT (Fig. 2c). Furthermore, IGF1R depletion attenuated AKT phosphorylation (Fig. 2b). The application of 1 μM picropodophyllin, a specific inhibitor of IGF1R, also attenuated AKT phosphorylation (Fig. 2b). Thus, in the medium, either IGF1 or insulin activates IGF1R, and its signal is transduced to AKT. Furthermore, the treatment of cells with 2DG up-regulates multiple signal transduction pathways [12], as noted in RCC4 cells (Fig. 2b). Zhou and colleagues suggested that 2DG up-regulates IGF1R by directly binding to its inhibitor, IGFBP3 [12]. However, using purified recombinant proteins, Pollak and colleagues showed that the binding between IGF1R and IGFBP3 is not disrupted by 2DG [13]. Thus, the molecular mechanism by which 2DG up-regulates multiple signaling pathways remains unresolved. What is clear from these data is that IGF1R is the major source of pro-survival signals in cultured RCC4 cells. One model explaining how the absence of VHL confers cell resistance to 2DG-ABT is that in the absence of VHL, IGF1R expression is up-regulated in RCC4 and UOK121 cells, thus generating a pro-survival signal through PI3K-AKT and causing these cells to be resistant to 2DG-ABT. In contrast, in RCC4 + VHL, UOK121 + VHL, and UOK121 + 5Aza cells, VHL interferes with IGF1R expression, attenuating the PI3K-AKT pro-survival signal and making these cells sensitive to 2DG-ABT. This possibility is supported by our observation that there were significant differences in the apoptotic rates of RCC4 cells, which express IGF1R, RCC4 + VHL cells, which express moderate amounts of IGF1R, and RCC4 cells depleted of IGF1R, which express almost no IGF1R, after treatment with 10 mM 2DG and 10 μM ABT-263 (Figs. 1 and 2).

### IGF1R activated both the Ras-ERK and PI3K-AKT pathways, but only the latter pathway interfered with mitochondria-dependent apoptosis

IGF1R, like EGFR and many other receptor tyrosine kinases (RTKs), activates both the Ras-ERK proliferation pathway and the PI3K-AKT pro-survival pathway. Activated Ras generates ERK signals through its association with Raf. Activated Ras also directly activates PI3K. Thus, there is cross-talk between these two pathways in the early stages of IGFR-induced signal transduction. However, after these signals reach PI3K and ERK, we can clearly distinguish between the activation of these two pathways. We were thus able to determine which of these two pathways interferes with the apoptotic activation of mitochondria.

**Fig. 3:**
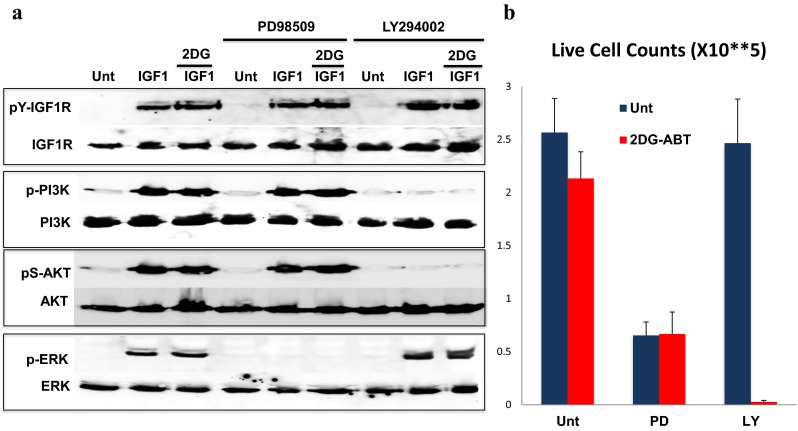
IGF1R activated both the Ras-ERK and PI3K-AKT pathways, but only the latter pathway interfered with mitochondria-dependent apoptosis. **a** Serum-starved UOK121 cells were stimulated with or without 20 ng/ml of IGF1 for 10 min in the presence or absence of either 50 μM PD98509 (ERK inhibitor) or 50 μM LY29402 (PI3K inhibitor). Cells were harvested and analyzed by western blotting using anti-phospho-IGF1R, anti-pS473-AKT, anti-P(T202/Y204)-ERK, and anti-IGF1R, PI3K, AKT, and ERK antibodies, as indicated. **b** UOK121 cells were first treated with or without 10 mM 2DG for 2 h, after which, either PD98509, LY294002, or DMSO was added for 5 min, prior to the addition of either 2 μM ABT-263 or DMSO for another 2 h. Then, the cells were washed, re-seeded in fresh media, and incubated overnight. The experiment was performed in triplicate, and live cells were counted the next day. The error bars indicate the standard deviation

To address this question, we used PD98509, a specific inhibitor for ERK1/2, and LY294002, a specific inhibitor for PI3K, in UOK121 cells, because in these cells, both Ras-ERK and PI3K-AKT activities are robust and easily observable. Pre-treatment of UOK121 cells with either inhibitor did not interfere with ligand-induced IGF1R activation, even in the presence of 2DG. However, PD98509 clearly blocked the activation of ERK without interfering with PI3K, whereas LY294002 specifically inhibited PI3K activation without interfering with ERK, clearly distinguishing the two pathways (Fig. 3a).

To induce apoptosis in UOK121 cells, we first pre-treated UOK121 cells with 10 mM 2DG for 2 h. Then, either the PI3K inhibitor or the ERK inhibitor was added for 5 min, prior to the addition of 2 μM ABT-263. Two hours later, the cells were washed and incubated in fresh medium overnight, until the cell viability assay was performed. In PD98509-treated cells, cell growth was arrested, but 2DG-ABT had little effect on survival (Fig. 3b). In stark contrast, LY294002-treated cells grew well, with approximately the same growth rate as untreated cells. However, with the additional treatment of 2DG-ABT for only 4 h, most of the cells underwent apoptosis, leaving very few surviving cells (Fig. 3b).

From these results, it appeared likely that in VHL-deficient cells, IGF1R expression is stabilized, thus generating PI3K-AKT pro-survival signals and interfering with the apoptotic activation of mitochondria. Accordingly, we investigated whether AKT interferes with the apoptotic activation of mitochondria by stabilizing the Bak-Mcl-1 association. To reduce AKT activity, we used both LY294002 and siRNA to lower the expression levels of isoforms 1 and 2.

### Inhibiting AKT sensitized cells to 2DG-induced dissociation of mcl-1 from Bak

**Fig. 4:**
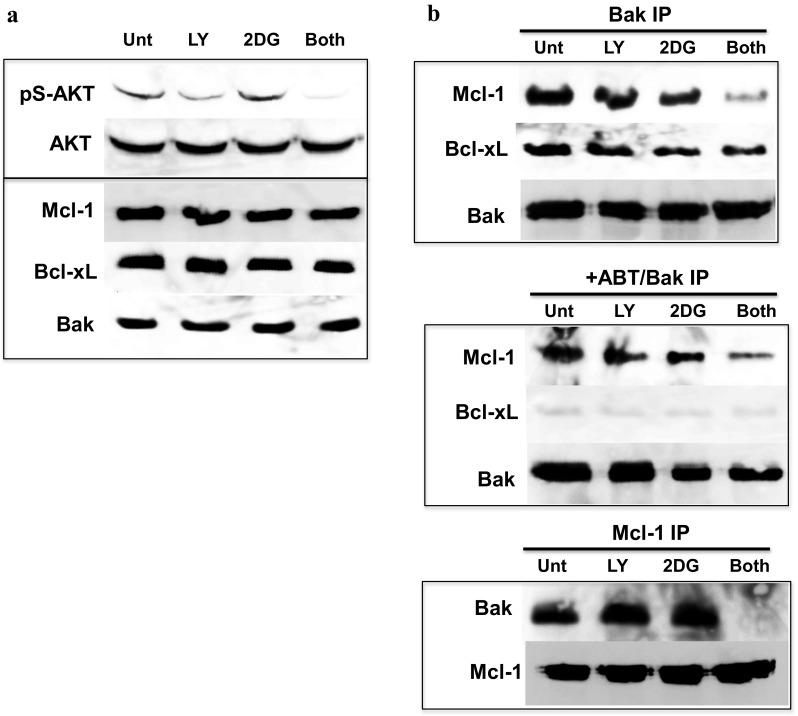
Inhibiting AKT sensitized UOK121 cells to 2DG-induced dissociation of Mcl-1 from Bak. Cells were treated with 10 mM 2DG, 50 μM LY294002, both, or left untreated for 4 h. The cells were harvested, and whole cell extracts were analyzed by western blotting **(a).** Some cells were also resuspended in immunoprecipitation buffer (see Materials and Methods section) and immunoprecipitated with anti-Bak antibody **(b**; *upper panel* **)**, anti-Bak antibody in the presence of 2 μM ABT-263 **(b**; *middle panel* **)**, or with anti-Mcl-1 antibody **(b**; *lower panel* **)**. The immunoprecipitates were analyzed by western blotting. For a preliminary experiment showing that ABT-263 could disrupt binding between Bak and Bcl-xL in vitro, see Sup Fig. 2

The PI3K inhibitor LY294002 did not alter the Bak-Mcl-1 association on its own (Fig. 4b), but in combination with 2DG, it significantly disrupted the Bak-Mcl-1 complex, almost completely dissociating Mcl-1 from Bak. These observations were made during the 4-h treatment period, in which there were no substantial changes in the levels of AKT and Mcl-1 (Fig. 4a). Thus, the kinase activity, and not the levels, of AKT must affect the stability of the Bak-Mcl-1 complex. The results from UOK121 cells are shown in Fig. 4. Similarly, in RCC4 cells, when the kinase activity of AKT was attenuated by blocking the signal transduction from PI3K to AKT, treatment with 2DG was sufficient to dissociate Mcl-1 from Bak [10]. Next, we tested whether lowering the protein levels of AKT could destabilize the Bak-Mcl-1 complex.

### Depleting AKT isoforms destabilized the Bak-mcl-1 complex and sensitized cells to 2DG-ABT-induced apoptosis

**Fig. 5:**
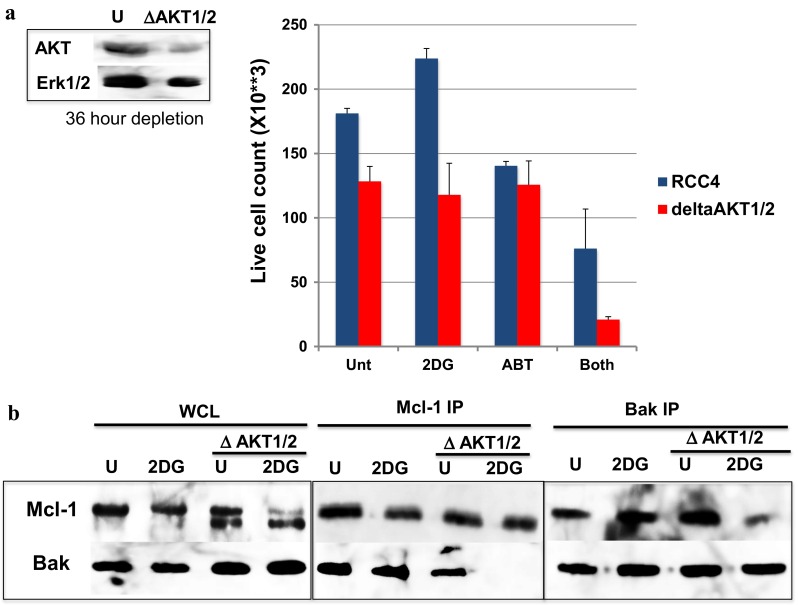
Partial depletion of AKT sensitizes RCC4 cells to 2DG-induced dissociation of Mcl-1 from Bak, thereby sensitizing cells to 2DG-ABT induced apoptosis. **(a**; *left panel* **)** AKT isoforms 1 and 2 were depleted from RCC4 with siRNA for 36 h, and the resulting cells are indicated as ΔAKT1/2. These cells and untreated cells were analyzed by western blotting. **(a**; *right panel* **)** These cells were assayed for 2DG-ABT-induced apoptosis, as in Fig. 3b. Live cells were counted, and the results are shown in the graphs. (**b;** WCL) RCC4 and ΔAKT1/2 were also incubated with 10 mM 2DG for 4 h, and the whole cell lysates analyzed by western blotting. (**b**; Mcl-1 IP and Bak IP panels). 2DG-treated and untreated cells were also re-suspended in immunoprecipitation buffer and incubated with anti-Mcl-1 or anti-Bak antibodies. The immunoprecipitates were analyzed by western blotting

Because AKT activates the transcription of Mcl-1, the prolonged absence of AKT may cause a loss of Mcl-1 expression, in which case, testing whether Mcl-1 associates with Bak would not be useful. We circumvented this problem by inactivating AKT with a PI3K kinase inhibitor and testing the Bak-Mcl-1 association within 2 h of AKT inhibition. At this time point, we observed minimal changes in Mcl-1 protein levels (Fig. 4). Although three known AKT isoforms are expressed in RCC4 [14], we depleted only two of them, isoforms 1 and 2, using siRNA in RCC4 cells. Our anti-AKT antibody used in this experiment recognizes all three isoforms of AKT, but because these isoforms are almost the same size, it is difficult to distinguish them. Nevertheless, after we treated cells with siRNA for 36 h, the overall expression levels of the AKT proteins were reduced (see ΔAKT1/2 in Fig. 5a). We also observed that the Mcl-1 proteins had altered mobility (compared with the mobility of the Mcl-1 bands in Fig. 5b), and the amounts of Mcl-1 were slightly reduced. To test whether the altered mobility was caused by caspases, we depleted AKT isoforms 1 and 2 in the presence of the pan-caspase inhibitor z-VAD. However, Mcl-1 still showed slightly increased mobility in SDS-PAGE gels (Sup Fig. 3). Thus, it appears likely that the Mcl-1 phosphorylation that occurs in the presence of AKT1/2 played a role in this mobility shift. Furthermore, because activated PI3K-AKT is known to up-regulate Mcl-1 [15], the reduced levels of Mcl-1 protein in AKT1/2-depleted cells may simply have been due to the reduced transcription of Mcl-1. Despite the reduced Mcl-1 levels, Bak and Mcl-1 co-immunoprecipitated in ΔAKT1/2 cells (Fig. 5b). However, in ΔAKT1/2 cells, 2DG readily dissociated Bak from Mcl-1 (Fig. 5b). When we quantified the number of cells 16 h later, ΔAKT1/2 cell growth was reduced compared with that of the untreated cells (Fig. 5a). However, more importantly, 2DG-ABT was substantially more effective in reducing the number of live ΔAKT1/2 cells compared with untreated cells. Thus, the most likely mechanism for the resistance of VHL-defective renal cancer cells to 2DG-ABT therapy is that in the absence of VHL, IGF1R expression is stabilized, thus activating AKT. Subsequently, the activated AKT interferes with the 2DG-induced Bak-Mcl-1 complex disassembly.

## Discussion

Following our publication on the 2DG-ABT combination treatment in 2011, the same combination has been tested on gastrointestinal stromal tumors (GIST) by Muhlenberg and colleagues [16]. Because these tumors have highly elevated glucose uptake, they are ideal candidates for 2DG-ABT combination therapy. Muhlenberg and colleagues also reported that the glucose-2DG combination, when used at an approximately 2:5 M ratio, works well to enhance ABT-induced apoptosis, thus corroborating our findings [3], as well as those of others [4]. In their article, however, how the treatment with 2DG enhanced ABT-induced apoptosis was not clarified. Initially, the authors focused on the effect of 2DG on the tyrosine kinase KIT and found that 2DG treatment inhibits KIT and KIT-dependent signaling, including PI3K-AKT, in some GIST cells. For example, incubating GIST-T1 cells in medium containing 2 mM 2DG and 6.8 mM glucose for 24 h diminished the phosphorylation of GIST and AKT. However, this finding was not consistent in GIST430 cells, in which a 24-h incubation with 2DG was sufficient to diminish KIT phosphorylation in the first experiment (Fig. 2a) but not in the second (Fig. 4b). Consequently, AKT phosphorylation was diminished in the first experiment but was substantially higher in the second experiment. It is interesting to note that GIST-T1 cells are sensitive to the KIT kinase inhibitor imatinib (IC50 = 42 nM) [17], whereas GIST430 cells are resistant to imatinib [18]. From our perspective, these results suggest that GIST430 cells might be resistant to the KIT inhibitor because they can activate the pro-survival AKT pathway in the absence of KIT activity. Unfortunately, these questions were not pursued in that article. When the authors combined 2DG with ABT, the apoptotic rates in the combination-treated cells were slightly higher than that in cells treated with 2DG or ABT alone. However, whether 2DG and ABT work synergistically to induce apoptosis was not addressed.

Muhlenberg and colleagues also examined other factors involved in apoptosis, such as Mcl-1, Bcl-2, Bcl-xL, and Bim. However, the authors observed only minor changes in the expression of these factors in response 2DG and did not discuss how 2DG may enhance ABT-induced apoptosis via these proteins. Last, in most of their in vitro experiments, 2DG was left in the medium for 24–144 h. These conditions do not translate well to animal experiments because injected 2DG does not stay in the circulation for more than several hours. Thus, unless 2DG is infused, cancer cells in animals are not likely to be exposed to 2DG for more than a few hours. Accordingly, 2DG treatment effectively reduced the viabilities of cultured GIST cells in 6-day cytotoxicity assays. However, when the authors treated mice xenografted with GIST-T1 cells by feeding them 1 g/kg of 2DG daily for 2 weeks, contrary to their expectation, the 2DG-treated tumors grew faster than untreated tumors. When they isolated the tumors from the untreated and 2DG treated mice, they found that 2DG treatment had no effect on KIT or its phosphorylation. Subsequently, the authors terminated their experiments without testing the 2DG-ABT combination in tumor-bearing mice. Using pharmacological doses of 2DG (40–100 mg/kg [2, 19]), 2DG-induced delays in tumor growth (tumor growth curve shifts along the *X*-axis) have been reported numerous times in all types of cancers with varied genetic background, including our own studies. Because GIST cells are known to have elevated glucose uptake, it would be of interest to know whether there in vivo resistance to 2DG is specific to GIST-T1 cells or whether it applies to all GIST cells.

Thus, we believe that it is important to design in vitro protocols to simulate what cancer cells in animals might experience. For this reason, we used an in vitro protocol in which the cells were washed within 4 h of 2DG application [3, 10]. However, even with these improvements, there will be differences in what cancer cells experience in vitro and in vivo.

Most cancer cells in a large tumor exist as a dense cluster of cancer cells in highly hypoxic conditions [20]. These cancer cells adapt to the hypoxic environment by activating hypoxia-induced factors [21]. Consequently, some members of the Bcl-2 family of proteins [22, 23] and the anti-apoptotic protein IAP-2 [24] are up-regulated, causing cell resistance to apoptosis (reviewed in Wouters et al. [25]). Furthermore, VHL-deficient cancer cells activate many of the same hypoxia-induced factors, even in the presence of normal levels of oxygen. Thus, before conducting the experiments in the present study, we hypothesized that the activation of hypoxia-induced factors causes VHL-deficient renal cancer cells to be resistant to 2DG-ABT. Contrary to our expectations, we found the following: (1) the rates of 2DG-ABT-induced apoptosis in RCC4 cells, with or without functional VHL, were not influenced by oxygen concentration; (2) the absence of VHL stabilized IGF1R expression independently of oxygen concentration; and (3) IGF1R-generated signals interfered with apoptotic signals in the mitochondria. These findings clearly demonstrated that hypoxia-activated VHL does not play a role in the 2DG-ABT-induced apoptotic pathway. It has also been reported that ABT induces apoptosis in hypoxic conditions just as effectively as in normoxic conditions [26]. We believe that ABT-induced apoptosis is independent of the oxygen concentration because, with the exception of Mcl-1, ABT binds to all members of the Bcl-2 family of proteins with high specificity and high affinity [5]. Hence, several fold differences in the levels of Bcl-2 family proteins do not influence the outcome.

Thus, we sought to identify other underlying reasons for the observed resistance and found that the IGF1R expression was stabilized in the absence of VHL. When the IGF1R protein was depleted by siRNA, the cells became sensitized to 2DG-ABT. When we analyzed which signal transduction cascades generated by IGF1R interfered with the 2DG-ABT-induced apoptotic pathway, we found that PI3K-AKT had profound effects on 2DG-ABT-induced apoptosis.

**Fig. 6:**
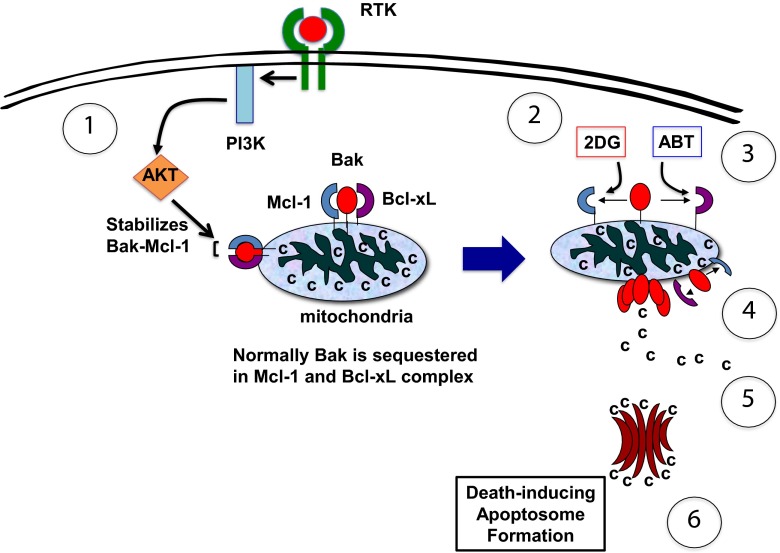
The model of signaling pathways in RTK-expressing cells. (*1*) In RTK-activated cells, AKT is activated by PI3K, stabilizing the Bak-Mcl-1 association in the mitochondria. (*2*) In the absence of activated AKT, 2DG can induce the dissociation of Mcl-1 from Bak. (*3*) ABT-263 binds to Bcl-xL with high affinity, dissociating it from Bak. (*4*) When freed from all its inhibitory associations, Bak is activated, forming a pore on the mitochondria. (*5*) Cytochrome c is released into cytosol. (*6*) The release of cytochrome c triggers the assembly of the death inducing apoptosome in the cytosol

AKT regulates multiple proteins involved in cell growth and survival, such as Cyclin D, GSK3B, Forkhead, mTOR, and BAD [27], and it is likely that one of these pathways activated by AKT may interfere with the 2DG-ABT-induced apoptotic pathway. By inhibiting GSK3B, which can phosphorylate and destabilize Mcl-1, AKT may potentially play a pro-survival role in 2DG-ABT induced apoptosis [28]. AKT also up-regulates Mcl-1 by activating its transcription. However, because 2DG-ABT induces apoptosis very quickly, releasing cytochrome c without altering Mcl-1 protein levels, these two pathways are not likely to be involved in the 2DG-ABT-induced apoptotic pathway. Upon closer examination, we found that a 4-h treatment of cells with the PI3K inhibitor LY294002 sensitized the Bak-Mcl-1 association, while maintaining the cellular protein levels of Mcl-1. Furthermore, attenuating AKT activity by inhibiting IGF1R or PI3K or by lowering AKT protein levels all lead to the destabilization of the Bak-Mcl-1 association. Thus, we concluded that in VHL-deficient cells, IGF1R activates AKT through PI3K, and AKT stabilizes the Bak-Mcl-1 complex, thereby making these cells resistant to mitochondria-dependent apoptosis. Accordingly, we were able to induce apoptosis in RCC4 or UOK121 cells (Fig. 6). First, we added 10 mM 2DG to the medium containing 25 mM glucose. Then, we lowered AKT activity by blocking either IGF1R or PI3K activity. Within 4 h, Mcl-1 was displaced from Bak. When we added ABT-263, which binds to all other Bcl-2 family members with high affinities, Bcl-xL was displaced from Bak. When freed from all its inhibitory associations, Bak is spontaneously activated, forming pores on the mitochondrial outer membranes, through which cytochrome c is released into the cytosol. In the cytosol, cytochrome c assembles apoptosomes, large protein complexes with protease activities. The apoptosomes are often called the death executioner because they cleave many proteins and are involved in the last step of the mitochondria-dependent apoptotic pathway.

One more issue remained to be addressed, namely, the molecular mechanism underlying the 2DG-induced dissociation of Mcl-1 from Bak. To this end, we identified a protein that binds to Mcl-1 when it is not associated with Bak. Currently, we are attempting to determine whether this protein binds competitively to Mcl-1, causing Bak-Mcl-1 dissociation, or whether it merely binds to Mcl-1 in the absence of the Bak-Mcl-1 association. We hope to report our new findings soon.

Applying the above concept to cancer therapies, however, would not be straightforward. First, a tumor mass is unlike the homogeneous cancer cells of a cell line. Due to tumor heterogeneity, some cells in a tumor may express IGF1R, whereas other cells may express EGFR, and yet other cells may express insulin receptors, and all these cells may generate PI3K-AKT pro-survival signals. If we were to use only an IGF1R receptor inhibitor, the cancer cells expressing EGFR or insulin receptors would not be eliminated, thus eventually causing tumor recurrence. Thus, AKT or at least, PI3K, must be inhibited. If we were to use LY294002, as we did in this article to treat mice bearing tumors, some mice would die from the treatment [29]. This result is because the PI3K-AKT pathway is activated in many healthy tissues, and most of its inhibitors have unwanted side effects. For this reason, we used beta-cyclodextrin (βCD) to interrupt the signaling between PI3K and AKT. The compound, βCD, is a reversible inhibitor of PI3K-AKT, and its effect in mice lasts only approximately 4 h and causes limited side effects. To take advantage of the absence of AKT, we needed to induce apoptosis very quickly. Therefore, we combined βCD with 2DG-ABT. The 2DG-βCD-ABT combination induced apoptosis in cultured UOK121 cells and also induced tumor regression in mice grafted with UOK121 cells. The results of the 2DG-βCD-ABT combination therapy have been reported [10].

Finally, it may still be possible to find a mild tyrosine kinase inhibitor that could attenuate AKT activity long enough to reduce Mcl-1 protein levels [30]. In some cells, 24 h might be enough to sufficiently reduce Mcl-1 protein levels such that ABT alone can induce apoptosis. In these scenarios, however, there is likely to be a trade-off between drug efficacy and toxicity [31].

## Materials and methods

### Reagents

Most of the experiments presented here were performed at least twice. Furthermore, some of the experiments were replicated in different cell lines but with different doses and time points, which were determined for each cell line in preliminary experiments.

The anti-P-PI3K antibody was obtained from Santa Cruz, the polyclonal anti-PI3K was from Pierce, the anti-HIF1a antibody was from BD Transduction Laboratories, the cytochrome c antibody was from BD Pharmingen (Cat. 556433 for blots), and the anti-β-tubulin antibody was from BD Bioscience. For pY-IGF1R, we used the anti-pY1135/1136 IGF1R antibody from CST (Cat#3024), and for pS-AKT, we used the anti-pSer 473 AKT1 antibody from Upstate (Cat#05-736). All other primary antibodies were purchased from Cell Signal (CST). Secondary antibodies conjugated with HRP were purchased from GE Healthcare. Most of these antibodies have been tested for specificity by us and other groups. ABT-263 was purchased from ChemieTek; 2-Deoxy-D-glucose was purchased from Sigma. The pan-caspase inhibitor z-VAD was purchased from Promega. Picropodophyllin was purchased from COSMO Bioscience. PD98509, an ERK inhibitor, was purchased from Merck, and LY29402, a PI3K inhibitor, was purchased from CST.

### Cell lines and cell culture

Renal cell carcinoma cell lines stably transfected with an empty vector or an expression vector encoding VHL were from the Harada Laboratory (Kyoto University Hospital, Dept. of Anesthesia, Japan), and UOK121 and UOK121 + VHL cells stably transfected with the VHL expression vector were kind gifts from Dr. Marston Linehan [9] (Center for Cancer Research, Urologic Oncology Branch, NCI). These cells were all cultured in high-glucose (4.5 g/L) DMEM supplemented with 10 % FBS, unless stated otherwise. To restore epigenetically silenced VHL expression in UOK121 cells, we cultured the cells in medium containing 5.0 μM 5-Aza-dCyd (Invitrogen) for 10 days [9]. The restoration of VHL expression and down regulation of IGF1R were verified by western blotting.

### Western blot and immunoprecipitation

We typically ran 20 μg of proteins per lane for the western blots. We usually ran the same set of samples on multiple gels so as to minimize the number of times we had to re-probe the membrane. The concentration of acrylamide/bis in the gels varied and was dependent on the size of proteins of interest. For immunoprecipitation, 200 μg of protein was resuspended in 20 mM Tris pH 7.5, 1 % Triton-X 100, 150 mM NaCl, 10 % glycerol, and phosphatase inhibitor cocktail from Cell Signaling (#58709S) and mixed with Protein G Sepharose or Protein A Sepharose (Sigma P3296/P9242) pre-conjugated with the antibodies for immunoprecipitation.

#### FACS analysis

After apoptosis was chemically induced, the cells were washed in PBS, re-incubated in regular medium and incubated overnight. A cell death assay was performed the next morning using the Propidium Iodide Incorporation assay. Cells were analyzed with BD Facs Canto II or FacsCalibur II, and the results were analyzed by FlowJo. These experiments were performed in triplicate for each condition, and the error bars indicate the standard deviations. Thus, for each FACS run presented in Sup Fig. 1, we analyzed over 50 samples, including controls.

#### IGF1R and AKT depletion

IGF1R protein was depleted from renal cancer cells using siRNA purchased from Cell Signaling (Cat 6610). The siRNA was transfected into renal cancer cell lines using Lipofectamine 2000 (Life Technologies), according to the manufacturer’s protocol. IGF1R depletion was confirmed by western blotting. AKT isoforms 1 & 2 were depleted from RCC4 cells using siRNA (Cat. 6621) from Cell Signaling, using the same protocol described above for depleting IGF1R.

### Live cell counts

Live cells were distinguished from dead cells by suspending the cells in trypan blue in PBS and counting them with an Invitrogen cell counter. These experiments were performed in triplicate.

### Statistical analyses

Statistical analysis was performed using GraphPad, http://www.graphpad.com/quickcalcs/ttest1.cfm? Format=C.

## Electronic supplementary material

ESM 1 (PDF 470 kb)
