# Supplementary material for: VHL-deficient renal cancer cells gain resistance to mitochondria-activating apoptosis inducers by activating AKT through the IGF1R-PI3K pathway
Source: Tumour Biol. 2016 Jul 26;37(10):13295–306. doi: 10.1007/s13277-016-5260-2 (PMC5097090; doi:10.1007/s13277-016-5260-2)
Supplement: Supplementary file 1 — (PDF 470 kb) [file 13277_2016_5260_MOESM1_ESM.pdf]

VHL deficient renal cancer cells gain resistance to mitochondria-activating apoptosis inducers by activating AKT though the IGF1R-PI3K pathway

Ryuji Yamaguchi [1,*], Hiroshi Harada[2,3], and Kiichi Hirota [1]

Supplemental Information

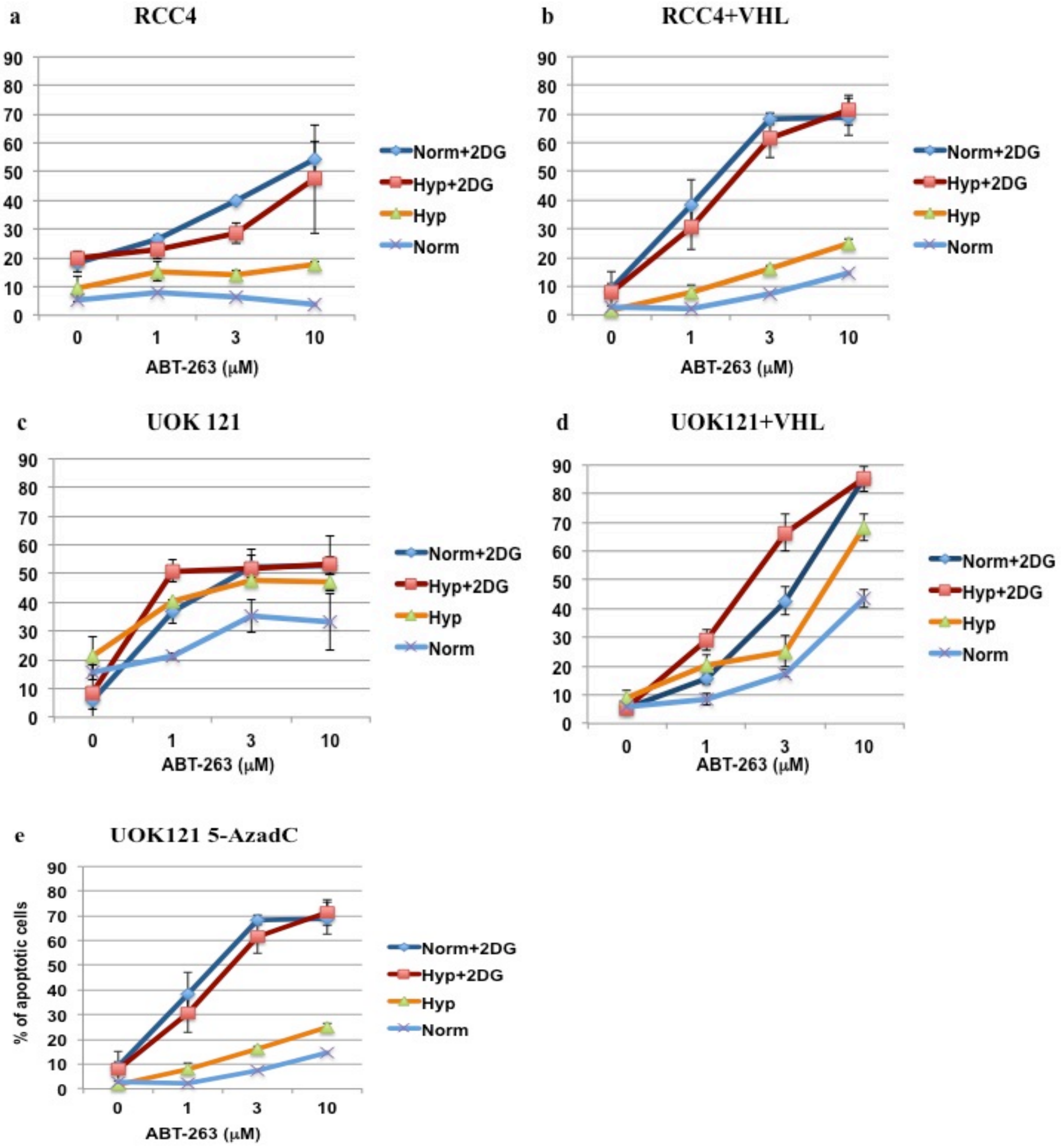

Supplemental Figure 1. The rates of apoptosis by 2DG-ABT were lower in VHL-deficient cancer cells under both normoxic and hypoxic conditions. (a-e) Renal cancer cell lines **(a)** RCC4, **(b)** RCC4+VHL, **(c)** UOK121, **(d)** UOL121+VHL and **(e)** UOK121 treated with 5-Aza-dC (UOK121+5-Aza-dC) to restore VHL expression, were all tested for sensitivity

to 2DG-ABT combination therapy; first with 10mM 2DG and then 2 hours later with ABT concentration varying from 0-10 μM. Then at 4 hours from the start of the combination treatment, all cells were washed and re-incubated in fresh media. Cells were assayed under either normoxic (21% oxygen) or hypoxic (1% oxygen) conditions, and analyzed for propidium iodide incorporation by FACS.

P-values for paired X^2 tests

	RCC4	RCC4+VHL	UOK121	UOK121+VHL
Norm vs Hypox	0.9627	0.6490	0.0375	0.0181
Unt vs 2DG	0.0009	0.0053	0.1742	0.0168

Supplemental Table 1. P-values were calculated from data presented in Sup Fig 1 a-d.

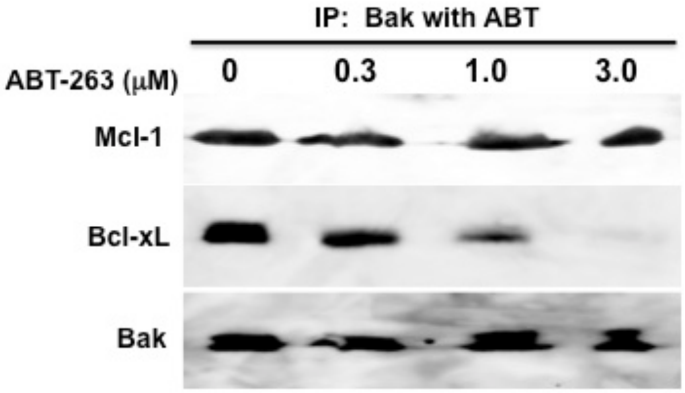

Supplemental Figure 2. Preliminary experiment: testing if ABT-263 in IP buffer could interfere with Bak-Bcl-xL association. Indicated amounts of ABT-263 was added to IP buffer containing untreated cells with Bak antibody conjugated with sepharose beads, as described in Materials and Methods.

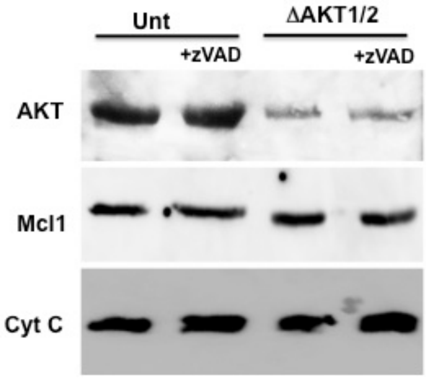

Supplemental Figure 3. The loss of AKT1/2 caused slight shift in Mcl-1 mobility in the presence and absence of the pan-caspase inhibitor zVAD. UOK121 cells were treated with siRNA to deplete AKT isoforms 1 & 2 for 30 hours in the presence and absence of zVAD. The sample was run together with indicated controls for western blots.
